# hsa-miR-518-5p/hsa-miR-3135b Regulates the REL/SOD2 Pathway in Ischemic Cerebral Infarction

**DOI:** 10.3389/fneur.2022.852013

**Published:** 2022-04-11

**Authors:** Boyan Zhao, Xiaofan Jiang

**Affiliations:** Department of Neurosurgery, Xijing Hospital, Fourth Military Medical University, Xi'an, China

**Keywords:** neurovascular, ischemic stroke (IS), scRNA-seq, regulatory network, REL

## Abstract

**Objectives:**

Ischemic cerebral infarction (ICI) is a fatal neurovascular disorder. A bioinformatics approach based on single-cell and bulk RNA-seq analyses was applied to investigate the pathways and genes involved in ICI and study the expression profile of these genes.

**Methods:**

First, the aberrantly regulated “small-molecule ribonucleic acids” [microRNA (miRNAs)] and messenger RNAs (mRNAs) were analyzed using transcriptome data from the ischemic brain infarction dataset of the Gene Expression Omnibus (GEO) database. In mouse cerebrovascular monocytes, the single-cell regulatory network inference and clustering (SCENIC) workflow was used to identify key transcription factors (TFs). Then, the two miRNA-TF-mRNA interaction networks were constructed. Moreover, the molecular complex detection (MCODE) extracted the core sub-networks and identified the important TFs within these sub-networks. Finally, whole blood samples were collected for validation of the expression of critical molecules in ICI.

**Results:**

We identified four cell types and 266 regulons in mouse cerebrovascular monocytes using SCENIC analysis. Moreover, 112 differently expressed miRNAs and 3,780 differentially expressed mRNAs were identified. We discovered potential biomarkers in ICI by building a miRNA-TF-mRNA interaction network. The hsa-miR-518-5p/hsa-miR-3135b/REL/SOD2 was found to play a potential role in ICI progression. The expression of REL and superoxide dismutase 2 (SOD2) was significantly elevated in the ICI group in the clinical cohort (*P* < 0.05). Furthermore, a REL expression was elevated in endothelial cells and fibroblasts at the single-cell level, indicating that REL is a cell-specific regulon. Functional enrichment analyses revealed that REL is primarily engaged in neurotransmitter activity and oxidative phosphorylation.

**Conclusions:**

Our research uncovered novel biomarkers for ICI of neurovascular disease. The hsa-miR-518-5p/hsa-miR-3135b may regulate the REL/SOD2 pathway in ICI progression.

## Introduction

Stroke is a common and fatal neurovascular disease that has high morbidity and mortality rates worldwide, accounting for ~17 million new cases annually (1–5). Ischemic stroke (IS) accounts for 80% of all stroke cases (6). Multiple emboli blocking the intracerebral arteries result in irreversible functional deficiencies in local brain tissue, eventually leading to ischemia and hypoxic necrosis (7, 8). Ischemic cerebral infarction (ICI) is a fatal neurovascular disorder (9). Due to its complexity, the molecular pathways underlying the development of ICI are not well-known at the transcriptome level. Exploring the regulatory network of signaling pathways is critical to understanding the mechanism by which ICI develops and to developing effective strategies for preventing and treating ICI.

Single-cell RNA sequencing (scRNA-seq) is a technique for obtaining whole-transcriptome expression profiles at the single-cell level. It is based on the amplification of microscopic whole-transcriptome RNA from isolated individual cells and subsequent high-throughput sequencing to elucidate the molecular regulatory mechanisms underlying specific biological processes and disease pathogenesis (10). In recent years, the scRNA-seq has gradually gained traction in the disciplines of oncology, microbiology, and neuroscience (11–13). In addition to studying changes in gene expression patterns at the population level, the scRNA-seq can be used to study single-cell gene expression, thus, resolving any bias arising due to cellular heterogeneity. Therefore, scRNA-seq is particularly well-suited for studying highly heterogeneous cell populations, such as neural cells (14, 15). Using scRNA-seq, Gate et al. showed that the T-cell receptor (TCR) signaling pathway is activated in CD8+ terminally differentiated effector cells (TEMRA) in the cerebrospinal fluid of patients with Alzheimer's disease, indicating that these cells contribute to the onset of neurological symptoms through their cytotoxic role (16). Vanlandewijck et al. used scRNA-seq to conduct a transcriptional investigation of the constituent cell types of the cerebral vasculature and discovered that endothelial cells, pericytes, and fibroblasts are implicated in the formation of neurovascular disease lesions. In this study, we attempted to address the dearth of molecular studies on cerebrovascular cell types and to establish the groundwork for future research on the molecular pathways underlying cerebrovascular disorders (17). The single-cell regulatory network infeAbegail Floresrence and clustering (SCENIC) is a computational approach for identifying cell states and constructing gene regulatory networks from scRNA-seq data (18). The SCENIC can be used to identify critical transcription factors (TFs) involved in the pathophysiology of ICI.

Apart from single-cell technology, various bioinformatics techniques have emerged as important tools to study complex biological phenomena. The “small-molecule ribonucleic acids” [microRNA (miRNAs)] are a class of non-coding, endogenous single-stranded RNA molecules composed of 20–24 nucleotides that regulate the expression of target genes in various physiological and pathological processes (19). The miRNAs serve as molecular markers for early diagnosis and prognosis, as well as therapeutic targets for ICI (20–23). The miR-PC-5P-12969 inhibits the production of amyloid and promotes IS (24). In acute ICI, the serum miR-124 and other miRs are inhibited, resulting in neuroinflammation and brain damage (25). Additionally, miRNAs can contribute to ICI by modulating TFs. Atherosclerosis (AS) has a significant role in the pathophysiology of ICI (26). Li et al. showed that miR-NA-663 governs the phenotypic metamorphosis of human vascular smooth muscle cells by adversely regulating the expression of its downstream TF, Jun B (27). The miRNA-23b can reduce vascular smooth muscle cell proliferation and migration, and the TF FoxO4 may be a direct target of miRNA-23b (28). Thus, the miRNAs play a critical role in the development of AS by controlling the proliferation, differentiation, and function of vascular smooth muscle cells *via* TF regulation, potentially altering the course of ICI. Therefore, single-cell sequencing is critical for identifying miRNA-TF-gene regulatory networks involved in ICI progression, which may reveal novel gene targets and molecular markers for ICI diagnosis and therapy.

In this study, we analyzed bulk RNA-seq, as well as scRNA-seq data, to identify a miRNA-TF-mRNA regulatory network that may be vital to ICI progression. Our research aimed to uncover novel biomarkers for ICI of neurovascular disease. The findings of this study may provide new avenues for the prevention and treatment of IS, as well as strategies to improve patient outcomes.

## Methods

### Data Acquisition

First, the ICI dataset was retrieved from the Gene Expression Omnibus (GEO) database. The GEO database is a repository of raw data, including transcriptomic data from published studies. From the GSE16561 dataset, transcriptomic data was acquired from whole blood samples of 39 patients with ICI, and 24 healthy individuals (29). Moreover, transcriptomic data from 24 control blood samples were collected from the GSE55937 dataset (30). The GSE98816, an scRNA-seq collection, contains transcriptomic information of 3,186 cells derived from mouse cerebrovascular tissue (17). Then, the expression of differentially expressed TFs in mouse cerebrovascular tissue was analyzed based on bulk RNA-seq data. Finally, to validate the expression of differentially expressed genes (DEGs) identified from bioinformatics analyses, whole blood samples were collected from 24 patients with ICI and 22 healthy individuals at our institution and were subjected to differential gene expression analysis. The inclusion criteria are: patients with ischemic cerebral infarction met the diagnostic criteria; patients with cerebral infarction were confirmed by imaging and the onset of the disease did not exceed 48 h; and informed consent was provided and signed by the patients. The exclusion criteria are: severe liver and kidney damage; cardiac diseases such as myocardial infarction and heart failure; acute complications of diabetes mellitus; acute infection combined with malignancy; and cerebral hemorrhage or cardiogenic cerebral embolism. This study was approved by the Institutional Ethics Review Board of The Xijing Hospital. Informed written consent was obtained from all the patients. The following statistical criteria were used for comparing differences between the two groups: ^*^P < 0.05, ^**^P < 0.01, and ^***^P < 0.001.

### scRNA-seq Analysis

The GSE98816, a scRNA-seq collection, was used for scRNA-seq analysis. The cells isolated from mouse cerebrovascular tissue were clustered and annotated to facilitate subsequent analysis of transcriptional regulation patterns in different cell populations. Like previous research, the R package Seurat (v. 4.0) was used to perform quality control on the scRNA-seq data, as well as data reduction, cell clustering, and cell annotation (31–33). After identifying highly variable genes using the “FindVariableFeatures” function, the principal component analysis (PCA) was performed to extract gene expression features from scRNA-seq data. The principal components (PCs) that account for the majority of the true signal were identified using JackStraw and Elbow plots. Then, cells were grouped according to these selected PCs. Cell clustering was performed using uniform manifold approximation and projection (UMAP). In the cell clustering results, the characteristic genes were identified for each cell cluster. Cell annotation was performed using SingleR (v. 1.0), and marker genes were annotated based on the results of previous studies (34, 35).

### SCENIC Analysis and Gene Regulatory Network Construction

Core TFs in scRNA-seq were obtained using the SCENIC method. The SCENIC is a computational approach for identifying cell states and constructing gene regulatory networks from scRNA-seq data (34). The SCENIC was used to analyze the significant TFs in each cell population after acquiring the annotation information and tissue origin of each cell. First, a gene co-expression network was constructed using data from a gene expression matrix of mouse cerebrovascular cells. Subsequently, from this gene co-expression network, the “RcisTarget” package in R was used to identify TF-mRNA regulatory networks with direct regulatory linkages, and these TFs were classified as regulons. Finally, a regulon activity score (RAS) was calculated for each cell corresponding to each regulon, which represents the regulatory activity of the associated regulon in each cell. Subsequently, these newly discovered regulons were used for further analysis.

### Differential Analysis and Identification of miRNA-mRNA Interactions

To identify genes that are dysregulated in IS, the “linear model” function of the “limma” package in R was used to compare the expression of mRNAs and miRNAs between the ICI and healthy control (HC) groups (36, 37). Differentially expressed mRNAs (DEmRNAs) and miRNAs (DEmiRNAs) were selected according to *P* < 0.05. Subsequently, the miRWalk (< http://mirwalk.umm.uni-heidelberg.de/>) open-source platform was used to identify DEmiRNA-DEmRNA networks.

### Identification of Molecular Interaction Networks and Core Regulons

First, the TF-target gene pairs present in the miRNA-mRNA network were predicted using the TRRUST (v2) database, followed by the identification of the key TFs in the network (38). Then, the core regulons were derived by conducting an intersection analysis on these major TFs, and the regulons were identified by SCENIC analysis. Finally, a network of interactions between miRNAs, TFs (regulons), and mRNAs were constructed. Protein-protein interaction (PPI) networks were visualized using Cityscape (v. 3.9), with all proteins corresponding to the nodes in this network. Furthermore, the Molecular Complex Detection (MCODE) plug-in was used to investigate and generate the core sub-networks of the gene interaction network (degree cutoff = 2, node score cutoff = 0.2, and maximum depth = 100).

### Gene Set Enrichment Analysis

To understand the function of the DEGs, the “clusterProfiler” package in R was used for functional enrichment analysis, including the Kyoto Encyclopedia of Genes and Genomes (KEGG) and Gene Ontology (GO) analyses (39). In contrast, the gene set enrichment analysis (GSEA) was performed to analyze the functional pathways involved in the screening of dysregulated regulons in patients with ICI (40). The reference gene set “c2.cp.v7.2.symbols.gmt [Curated]” was obtained from MSigDB Collections (https://www.gsea-msigdb.org/gsea/msigdb/), and *P*-values were calculated using a random sample swap (*n* = 1,000). The threshold for screening functional pathways was set as follows: false discovery rate (FDR) <0.25 and adjusted *P*-value <0.05.

### Quantitative Real-Time PCR

The total RNA was extracted from whole blood samples using TRIzol reagent (Invitrogen) according to the manufacturer's instructions. The RNA was reverse-transcribed to complementary DNA (cDNA) using a cDNA synthesis kit (K1622; Fermentas, Waltham, MA), following the manufacturer's instructions. Finally, quantitative real-time PCR (qRT-PCR) was performed using an ABI 7500 real-time PCR system (Applied Biosystems, CA, USA), and amplification was performed using SYBR Green (Prime Script RT Master Mix, Takara, Tokyo, Japan). The Glyceraldehyde-3-phosphate dehydrogenase (*GAPDH*) gene was used as an internal reference gene for the normalization of gene expression levels. As with previous research, the following gene-specific primers were used: *GAPDH*, forward: 5′-AGAAGGTGGTGAAGCAGGC-3′ and reverse: 5′-TCCACCACCCAGTTGCTG-3′; *REL*, forward: 5′-GAATCAATCCATTCAATGTCCC-3′ and reverse: 5′-AAGAGCAGTCGTCAAATTACC-3′; *SOD2*, forward: 5′-GACAAACCTCAGCCCTAACG-3′ and reverse: 5′-CGTCAGCTTCTCCTTAAACTTG-3′ (41, 42). The gene expression levels were calculated using the 2 ΔΔCT method and normalized to the mRNA levels of *GAPDH*.

### Statistical Analysis

Statistical analysis and graphing were performed using R software (version 4.0.2). Gene regulatory networks based on SCENIC analysis were constructed using the pySCENIC (v. 0.11) package in Python. The Pearson's correlation analysis was performed to calculate the correlation between two measured variables. Statistical significance was set at *P* < 0.05.

## Results

### Single-Cell Data Filtering and Cell Fractionation

**Figure 1:**
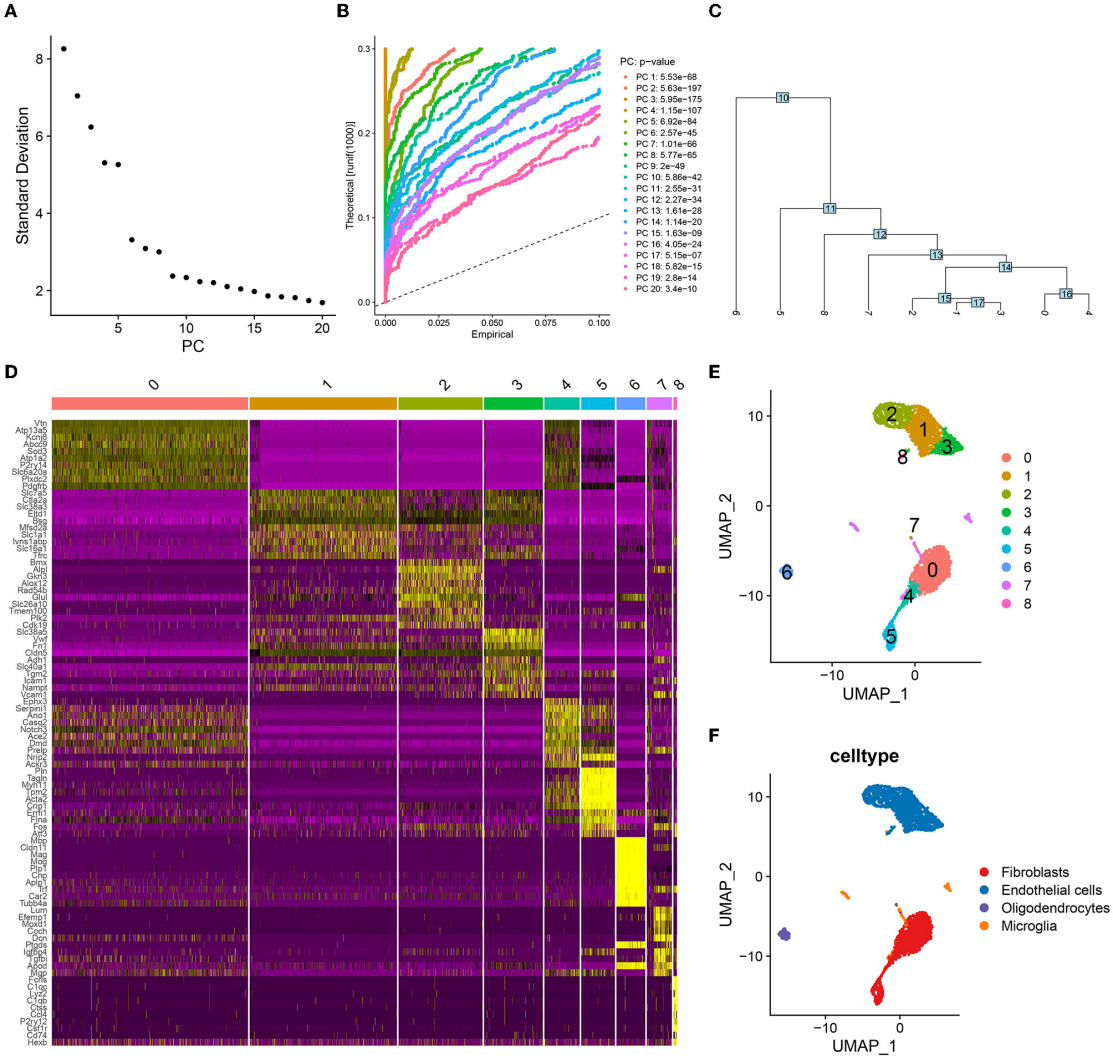
Cell clustering and annotation processes in single-cell analysis. **(A)** The top twenty principal components (PCs) were ranked according to the extent of the explanatory difference between them. According to the calculation, several PCs between 10 and 20 may cover all features of ICI. Here, 15 PCs are selected for the following round of investigation. **(B)** The top 20 PCs were found to be significantly enriched for features with low P-values. **(C)** A tree was constructed during the cell-clustering phase and nine distinct cell clusters were obtained. **(D)** The heat map shows the significant differences in the expression of marker genes in these nine cell clusters. **(E)** UMAP visualized the distribution of nine cell clusters. **(F)** These four cell clusters were annotated as fibroblasts, endothelial cells, oligodendrocytes and microglia, respectively.

First, we filtered the mouse cerebrovascular scRNA-seq data according to a set threshold (i.e., traits expressed in a minimum of 3 cells and cells with a minimum of 500 traits expressed were screened for inclusion in the study). A total of 3,186 cells were obtained for subsequent analysis. We estimated the top 2,000 DEGs and analyzed their expression patterns using PCA. The top 10–20 PCs were selected (Figure 1A). The JackStraw plot also verified that the top 20 PCs were enriched in most of the features with low *P*-values (Figure 1B). Fifteen PCs were selected for further clustering analysis, the results of which revealed nine-cell clusters (Figure 1C). The expression of the marker genes for these nine-cell clusters is depicted as a heat map in Figure 1D. The UMAP diagram depicts the clustering of these nine-cell clusters (Figure 1E). Based on the results of singleR and cell markers, the cell types were further classified as fibroblasts, endothelial cells, oligodendrocytes, and microglia (Figure 1F). Subsequently, we used SCENIC to identify 266 regulons, as well as the accompanying gene regulatory networks in four cell groups.

### miRNA-TF-mRNA Interactions Analysis

**Figure 2:**
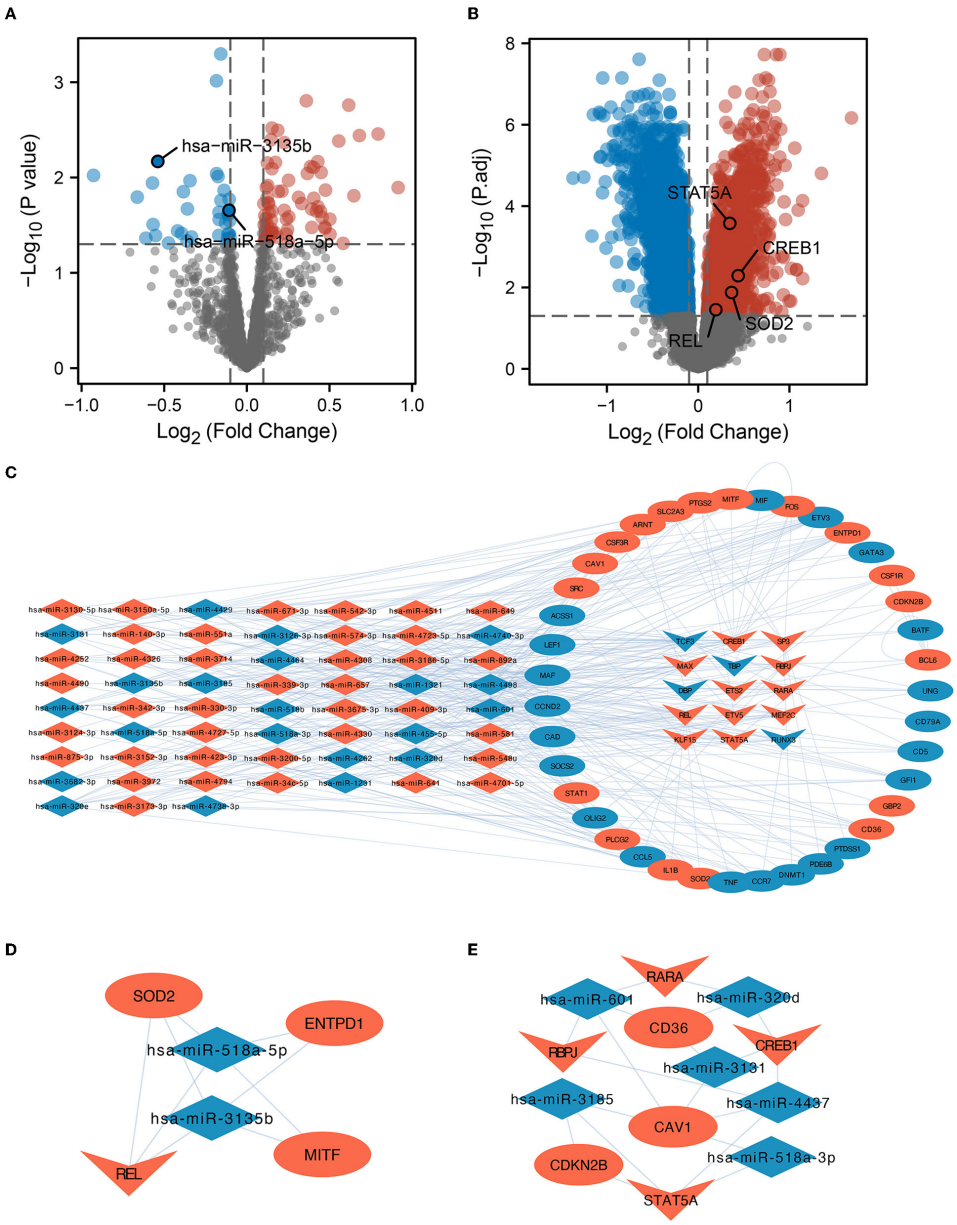
Variance analysis and PPI network construction. **(A)** A map of differentially expressed micro RNAs (DEmiRNAs) in the form of a volcano. The important miRNAs were identified and tagged. **(B)** Differentially expressed messenger RNA (DE-mRNA) volcano map. The diagram labels important transcription factors (TFs) and genes in the protein-protein interaction (PPI) sub-network. **(C)** Network investigation of miRNA-TF-gene connections. The network diagram depicts the connections between dysregulated miRNAs, TFs, and target genes. Dysregulated microRNAs are represented by the diamond, dysregulated genes are represented by the oval, and transcription factors are represented by the inner quadrilateral. Increased regulation is shown by the color red, whilst lower regulation is indicated by the color blue. **(D)** REL is the core TF in the PPI network. Subnetwork 1 of PPI and REL/superoxide dismutase 2 (SOD2) was regulated by the hsa-miR-518a-5p and hsa-miR-3135b microRNAs. **(E)** RARA, CREB1, RBPJ, and STAT5A were shown to be the most critical TFs in PPI Subnetwork 2.

Bulk RNAs were analyzed for differences in their expression between the ICI and HC groups based on GSE16561 and GSE55937. We found 73 upregulated and 39 downregulated miRNAs (Figure 2A), as well as 1,496 upregulated and 2,284 downregulated mRNAs, based on the filtering conditions (Figure 2B). Moreover, we found 11,525 DEmiRNA-DEmRNA interaction pairs using the prediction results of the miRWalk database. Based on the prediction results of the TRRUST database and the 266 regulons identified by SCENIC analysis, we identified 20 regulons and their corresponding target genes. Finally, the identified DEmiRNAs, DETFs, and DE gene mRNAs were used to build the miRNA-TF-gene interaction network (Figure 2C). Using MCODE, two key sub-networks (Figures 2D,E) were identified in this network. The REL was identified as the key TF in sub-network 1, while *SOD2* was identified as a target gene of REL (Figure 2D). Therefore, the hsa-miR-518a-5p and hsa-miR-3135b were found to control the expressions of *REL* and *SOD2*. Furthermore, TFs [Retinoic Acid Receptor Alpha (RARA), Recombination Signal Binding Protein For Immunoglobulin Kappa J Region (RBPJ), CAMP Responsive Element Binding Protein 1 (CREB1), and Signal Transducer and Activator of Transcription 5A (STAT5A)] and microRNAs (hsa-miR-601, hsa-miR-320d, hsa-miR-3131, hsa-miR-4437, hsa-miR-518-3p, and hsa-miR-3185) co-regulate mRNAs (CD36, CAV1, and CDKN2B) in sub-network 2 (Figure 2E). Here, potential mechanisms of ICI are uncovered based on miRNA-TF-mRNA integration analysis.

### Functional Pathways Enriched in the Core Network

**Figure 3:**
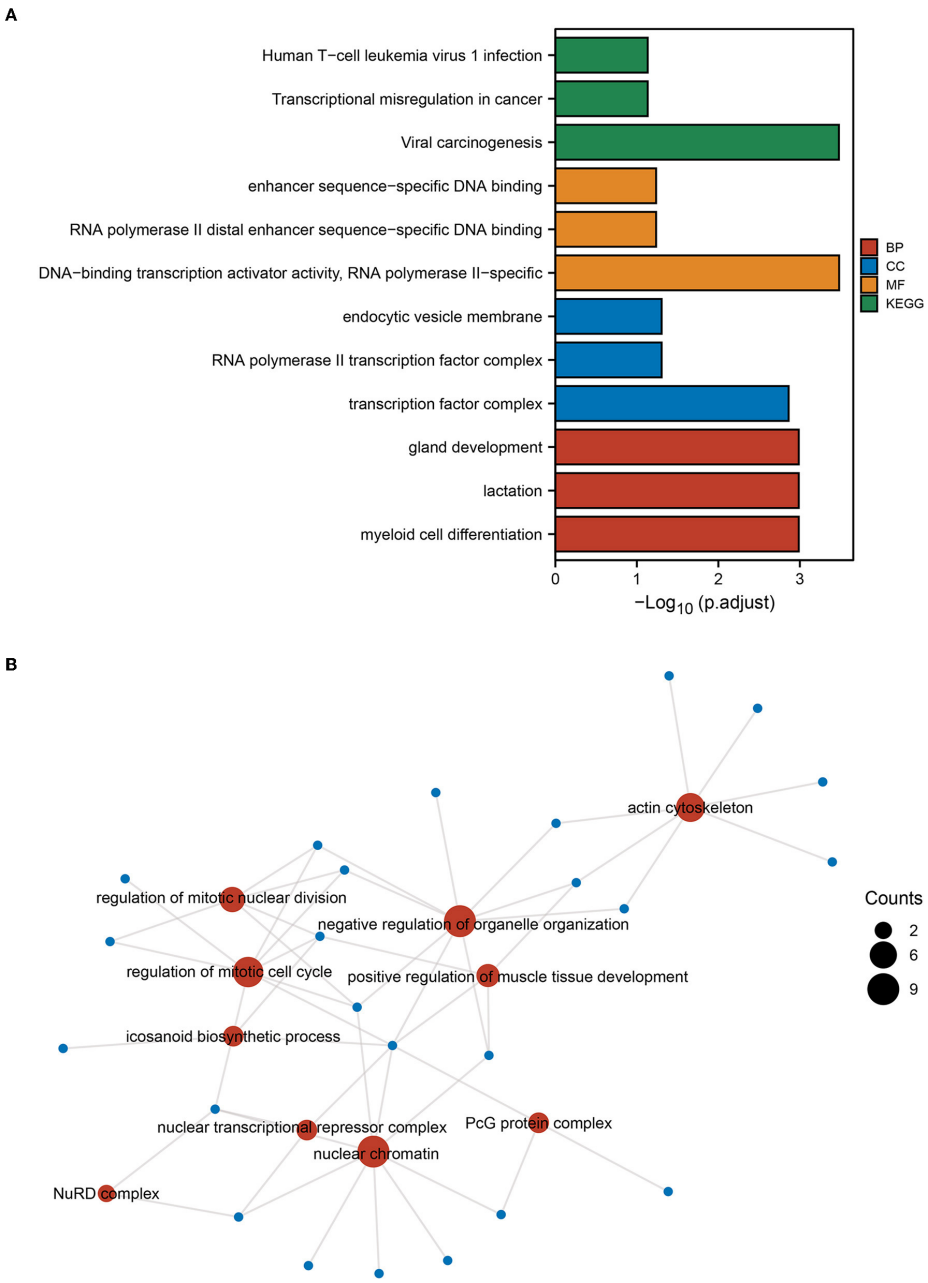
GO and KEGG enrichment analysis. **(A)** Gene Ontology (GO) and Kyoto Encyclopedia of Genes and Genomes (KEGG) enrichment analysis of genes from PPI subnetwork 1 and subnetwork 2 gene. **(B)** Based on the results of the single-cell regulatory network inference and clustering (SCENIC) analysis, GO functional enrichment analysis was performed for genes downstream of the transcription factor REL. The size of the dot indicates the number of genes.

Next, enrichment analysis of mRNAs in sub-networks 1 and 2 was conducted to investigate the pathways involved in the core sub-network. The findings revealed that the ICI group had increased functions and pathways involved in viral oncogenesis, DNA-binding transcriptional activator activity, transcription factor complex, and myeloid cell differentiation (Figure 3A). Then, we performed a functional enrichment analysis of the downstream target genes of the transcription factor REL based on the findings of SCENIC prediction. The findings revealed that the downstream molecules of *REL* were mostly enriched in the functional pathways of the actin cytoskeleton, positive control of muscle tissue development, eicosanoid biosynthetic process, and mitotic nuclear division regulation (Figure 3B). The results of the above enrichment analysis exemplify the potential functions and pathways enriched by these TFs and guide the way for further research.

### Expression of REL in Mouse Cerebrovascular Cells

**Figure 4:**
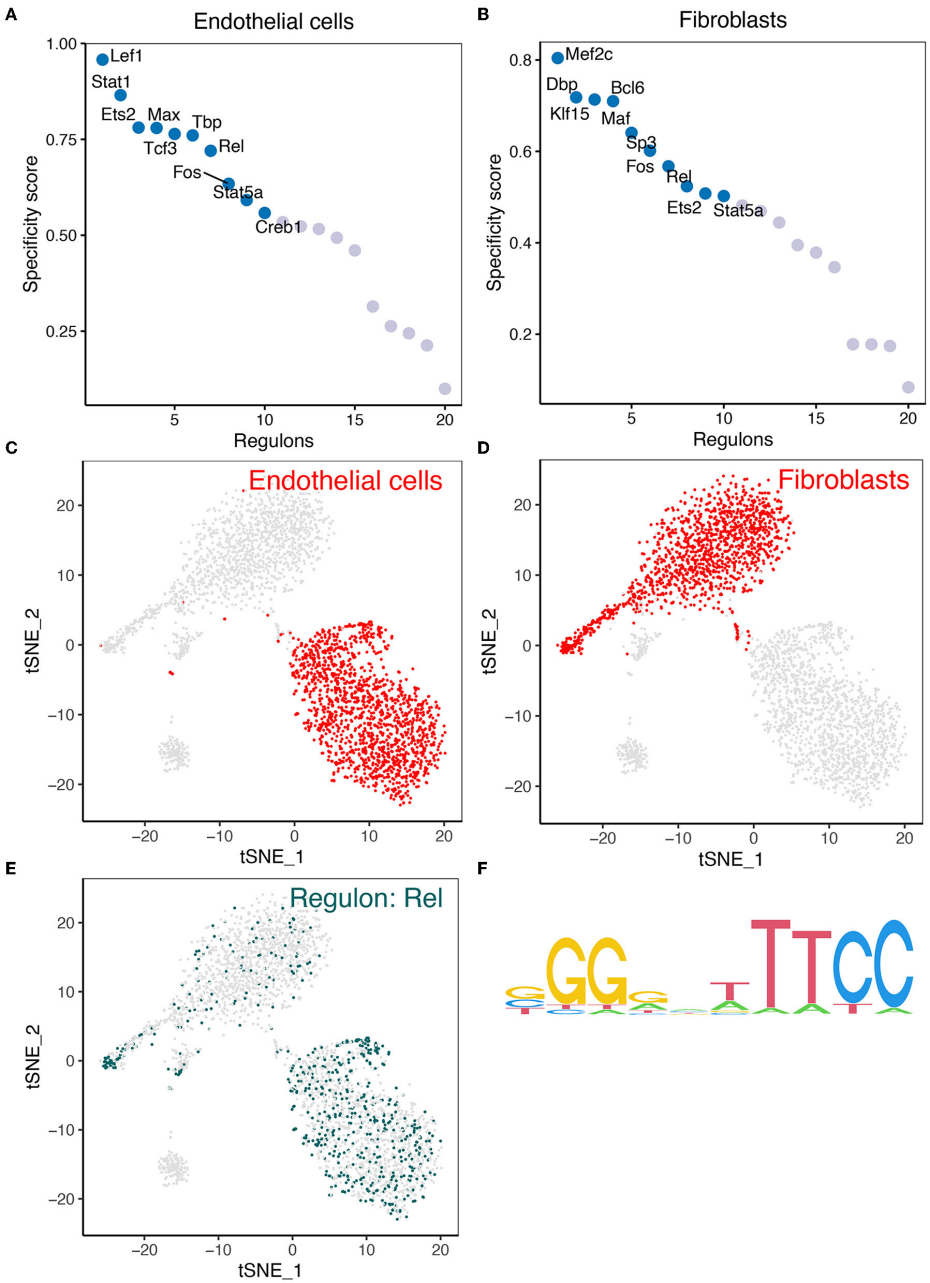
The findings of the SCENIC investigation of REL. **(A)** The regulon specificity score was used to rate the various regulators in endothelial cells. **(B)** The regulon specificity score was used to rank the various regulators in fibroblasts. **(C)** In the tSNE map, endothelial cells are visible. **(D)** Fibroblasts are visualized in the tSNE map. **(E)** *REL* was discovered to be significantly expressed in endothelial cells and fibroblasts. As can be seen, endothelial cells have greater levels of REL expression than fibroblasts. **(F)** Base distribution in REL-functioning motifs.

We analyzed the expression of key regulons in the gene interaction network in mouse cerebrovascular cells. Firstly, TFs in endothelial cells and fibroblasts were sorted for presentation according to the regulon specificity score (RSS) (Figures 4A,B). The t-Distributed Stochastic Neighbor Embedding (tSNE) plots in Figures 4C,D show the distribution of REL expression in endothelial cells and fibroblasts. Furthermore, the expression of *REL* is presented in the tSNE plot (Figure 4E). Figure 4F depicts the motifs of the downstream target genes of *REL*. The results revealed that REL is highly expressed in both endothelial cells and fibroblasts. However, *REL* is potentially regulated by hsa-miR-518-5p/hsa-miR-3135b (Figure 2D). We concluded that hsa-miR-518-5p/hsa-miR-3135b-regulated *REL*/*SOD2* plays a potential role in ICI progression.

### Expression of REL/SOD2 in Clinical Cohorts

**Figure 5:**
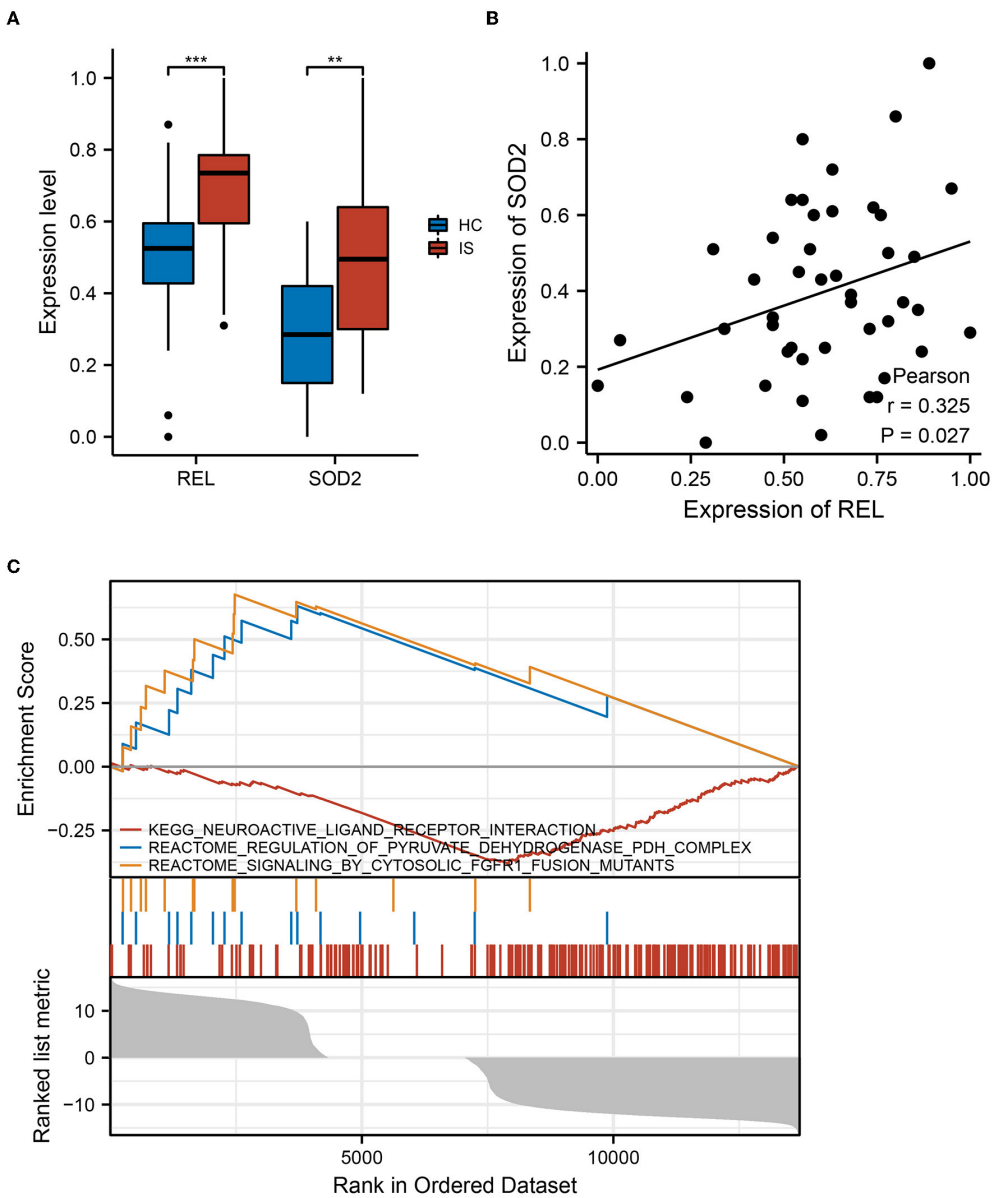
REL and SOD2 expression in clinical cohorts. **(A)** There was a substantial difference in REL and SOD2 expression between the healthy controls (HC) and Ischaemic cerebral infarction (ICI) groups. The ICI group had greater levels of REL and SOD2 expression than the HC group, and the results were statistically significant (*P* = 0.05). **means the *p*-value is less than 0.01, ***means the *p*-value is less than 0.001. **(B)** Pearson correlation analysis revealed a strong positive correlation between SOD2 and REL expression (*r* = 0.325, *P* = 0.027). **(C)** In samples with high REL expression, gene set enrichment analysis (GSEA) reveals up-and downregulated functional pathways.

The qRT-PCR was performed on blood samples from ICI and HC to further validate the results of the bioinformatic analysis in a clinical cohort. The expressions of *REL* and *SOD2* were significantly higher in the ICI group than in the HC group (*P* < 0.05) (Figure 5A). Additionally, results of PCA revealed a positive correlation between *SOD2* and *REL* expressions (*r* = 0.325, *P* = 0.027) (Figure 5B). These findings confirm that *REL* and *SOD2* are dysregulated in the ICI group.

### GSEA in Samples With Upregulated *REL* Expression

The GSEA was performed to further explore the pathways associated with *REL* expression. The results revealed that neuroactive ligand-receptor interaction and pyruvate dehydrogenase (PDH) complex regulation pathways were upregulated in samples with increased *REL* expression, but the cytosolic Fibroblast growth factor receptor 1 (FGFR1) fusion mutant signaling pathway was downregulated (Figure 5C). These findings imply that REL may be associated with the action of numerous neurotransmitters and their receptors, thus, affecting neurological and psychiatric symptoms. Additionally, REL may be involved in changes in oxidative phosphorylation, which is associated with ICI.

## Discussion

The major miRNA-TF-mRNA regulatory network in the ICI was investigated employing a bioinformatic approach that integrated single-cell analysis with bulk RNA-seq data. This study uncovered novel biomarkers for ICI of neurovascular disease. The hsa-miR-518-5p/hsa-miR-3135b/REL/SOD2 was identified as a potential player in ICI progression. The expression of the key molecules was also validated in clinical samples and at the single-cell level.

In this study, we first tried to construct two miRNA-TF-mRNA interaction networks and then found that REL was the key transcription factor. A miRNA-TF-mRNA interaction network was established and then two major sub-networks from this network were constructed. We also uncovered novel biomarkers for ICI of neurovascular disease. The hsa-miR-518-5p/(hsa-miR-3135b)/REL/SOD2 was identified as a key pathway involved in the course of ICI in sub-network 1. The RARA, CREB1, RBPJ, and STAT5A were identified as TFs involved in ICI progression in another network sub-network (sub-network 2). The REL and SOD2 expression were higher in the ICI group than in the clinical cohort. The REL expression was also enhanced in endothelial cells and fibroblasts at the single-cell level, indicating that it is a cell-specific regulon. Functional enrichment analyses revealed that REL is implicated in neurotransmitter activity and oxidative phosphorylation pathways.

No previous study has elucidated the direct role of miRNA-518 and miR-3135b in ICI. The hsa-miRNA-518 and its target gene Early growth response factor 1 (*EGR1*) are implicated in the regulation of cell proliferation, death, and angiogenesis (43). The *EGR1* serves as a pro-inflammatory factor in experimental animal models and is implicated in post-ischemic inflammation, inflammation-induced blood-brain barrier (BBB) dysfunction, and ischemic brain injury, suggesting that it plays a role in ICI progression (44–47). The peroxisome proliferator-activated receptor alpha (*PPAR*) mRNA may also be targeted by miR-518 (48). The PPAR suppresses the expression of the pro-inflammatory factor EGR1, which is important for lipid homeostasis and inflammation (44, 49). In a model of experimental IS, the PPARic stroke exped the (ul13) infarct volume (43, 44). The miR-518 may play a role in the evolution of ICI by regulating PPAR and EGR1. Human endothelial cells exposed to acrolein, an unsaturated aldehyde capable of generating oxidative stress and inflammation, showed higher expression of miR-518 (50). As a result, we postulate that miRNA-518 is implicated in IS-related inflammation and vascular endothelial dysfunction. In individuals with severe hypertension, miR-3135b expression was found to be dramatically increased (51, 52). Additionally, miR-3135b is linked to coronary artery calcification and is an excellent marker for the diagnosis of obstructive coronary artery disease (53). In contrast, diabetes, hypertension, atherosclerosis, and other metabolic disorders are all risk factors for ICI (26, 54–56). As a result, we postulate that miR-3135b has a role in ICI as well. Moreover, based on the increased expression of REL and SOD2 in patients with ICI compared to healthy controls, we postulated that hsa-miR-518-5p and hsa-miR-3135b were downregulated in ICI. The hsa-miR-518-5p and hsa-miR-3135b, which are differentially expressed in IS, are implicated in ICI progression *via* the regulation of the core TF, REL, and its target gene *SOD*2 in the ICI regulatory network. Therefore, based on our study and previous studies showing the significance of miR-518 and miR-3135b in IS-related pathogenic processes, we suggest that both may affect the development of ICI through various pathways.

The miRNAs can form gene regulatory networks by targeting TFs (57). The miRNAs may contribute significantly to the development of AS by controlling the proliferation, differentiation, and function of vascular smooth muscle cells through TF regulation, thereby affecting the course of ICI (26–28). Similar to previous findings, the current study showed that hsa-miR-518-5p and hsa-miR-3135b regulate the expression of their target gene *SOD2* by targeting the core TF REL, which forms the key regulatory network for ICI development. In this study, REL and SOD2 were highly expressed in patients with cerebral infarction at the bulk RNA, single-cell RNA, and clinical levels. Previous research indicates that v-rel reticuloendotheliosis viral oncogene homolog A (RELA) may enhance inflammatory responses and apoptosis in cerebral hemorrhage, however, c-REL may exert the opposite effect (58). Additionally, RELA, RELB, and c-REL, all of which are involved in the production of pro-inflammatory cytokines, are related to NF-κB-mediated inflammatory disorders (59). Inhibition of NF-κB activation prevented cerebral ischemia/reperfusion injury in rats (60). Inflammation is a risk factor for ICI and is associated with poor patient prognosis (61, 62). Therefore, we postulate that REL may affect the course of ICI and, hence, affect patient prognosis. Similarly, one study discovered that SOD2 is a mitochondrial enzyme with increased mRNA expression in patients with type 2 diabetes mellitus and those with Parkinson's disease (63). The SOD2 is involved in a variety of biological processes, including inflammation, glucose metabolism, and lipid metabolism, all of which contribute to the development of ICI (64–66). In this study, *SOD2* was identified as a target gene of REL in the ICI gene regulatory network. We found that REL might be involved in the activity of various neurotransmitters and their receptors, which may affect neurological and psychiatric symptoms. Therefore, we postulate that REL and ROS contribute significantly to ICI progression.

Along with the major sub-network hsa-miR-518-5p/hsa-miR-3135b/REL/SOD2, we identified a sub-network containing the transcription factors RARA, CREB1, RBPJ, and STAT5A. Previous studies have found that activation of RARA can exert neuroprotective effects after ischemic brain injury. Tanka et al. found a significant increase in phosphorylated CREB levels in the peri-infarct region after focal cerebral ischemia (67). The RBPJ is an important transcription factor of the Notch signaling pathway (68). Activation of the Notch signaling pathway is associated with atherogenesis and inflammatory responses, which are risk factors for ICI (26, 69, 70). After cerebral ischemia, activated STAT5 could improve the hypoxia tolerance of neurons and inhibit apoptosis, as well as participate in the protective effect of brain tissue (71). Similar to previous studies, we found that RARA, CREB1, RBPJ, and STAT5A may be critical transcription factors implicated in the development of ICI.

We studied the miRNA-TF-gene interaction network (hsa-miR-518-5p/hsa-miR-3135b regulates the REL/SOD2 pathway), which may play a critical role in ICI progression. Our research uncovered novel biomarkers for ICI of neurovascular disease. The findings of this shed light on the mechanism of ICI development and are expected to facilitate the identification of novel targets and therapeutic strategies for the treatment of ICI. There are certain limitations to the present study. In the future, the sample size for inclusion in the study should be further expanded. Since the findings of this study are mostly based on bioinformatic analysis, the identified signaling pathways still require experimental validation, including elucidation of their mechanisms of action, *in vitro* and *in vivo*.

## Conclusion

Our research uncovered novel biomarkers for ICI of neurovascular disease. The REL/SOD2 pathway is important in the formation of ICI as a key network route and may be regulated by hsa-miR-518-5p/hsa-miR-3135b. This study's findings provide insight on the potential relevance of the REL/SOD2 pathway in IS, paving the way for additional research into ICI mechanisms.

## Data Availability Statement

Publicly available datasets were analyzed in this study. This data can be found here: National Center for Biotechnology Information (NCBI) Gene Expression Omnibus (GEO), https://www.ncbi.nlm.nih.gov/geo/, GSE16561, GSE55937 and GSE98816.

## Ethics Statement

The studies involving human participants were reviewed and approved by the Institutional Ethics Review Board of the Xijing Hospital. Written informed consent to participate in this study was provided by the patient/participants' or patient/participants legal guardian/next of kin.

## Author Contributions

BZ: software, validation, formal analysis, data curation, and writing—original draft. XJ: conceptualization, methodology, supervision, writing—review and editing, and project administration. Both authors contributed to the article and approved the submitted version.

## Conflict of Interest

The authors declare that the research was conducted in the absence of any commercial or financial relationships that could be construed as a potential conflict of interest.

## Publisher's Note

All claims expressed in this article are solely those of the authors and do not necessarily represent those of their affiliated organizations, or those of the publisher, the editors and the reviewers. Any product that may be evaluated in this article, or claim that may be made by its manufacturer, is not guaranteed or endorsed by the publisher.

## Abbreviations

AS: atherosclerosis
IS: ischemic stroke
scRNA-seq: single-cell RNA-sequencing
SCENIC: single-cell regulatory network inference and clustering
SOD2: superoxide dismutase 2
TF: transcription factor.

## References

[1] Guo M Wang X Zhao Y Yang Q Ding H Dong Q. Ketogenic diet improves brain ischemic tolerance and inhibits NLRP3 inflammasome activation by preventing Drp1-mediated mitochondrial fission and endoplasmic reticulum stress. Front Mol Neurosci. (2018) 11:86. 10.3389/fnmol.2018.00086 29662437 PMC5890101

[2] Zhao B Shi Q Zhang Z Wang S Wang X Wang H. Protective effects of paeonol on subacute/chronic brain injury during cerebral ischemia in rats. Exp Ther Med. (2018) 15:3836–46. 10.3892/etm.2018.5893 29563983 PMC5858057

[3] Zheng Z Liu S Wang C Wang C Tang D Shi Y. Association of genetic polymorphisms in CASP7 with risk of ischaemic stroke. Sci Rep. (2019) 9:18627. 10.1038/s41598-019-55201-y 31819117 PMC6901581

[4] Bonita R Mendis S Truelsen T Bogousslavsky J Toole J Yatsu F. The global stroke initiative. Lancet Neurol. (2004) 3:391–3. 10.1016/S1474-4422(04)00800-2 15207791

[5] Weinstein JR Koerner IP Möller T. Microglia in ischemic brain injury. Fut Neurol. (2010) 5:227–46. 10.2217/fnl.10.1 20401171 PMC2853969

[6] Shang Y Chen L Toborek M Yu G. Diffuse optical monitoring of repeated cerebral ischemia in mice. Opt Express. (2011) 19:20301. 10.1364/OE.19.020301 21997041 PMC3495871

[7] Favate AS Younger DS. Epidemiology of ischemic stroke. Neurol Clin. (2016) 34:967–80. 10.1016/j.ncl.2016.06.013 27720004

[8] Benjamin EJ Virani SS Callaway CW Chamberlain AM Chang AR Cheng S. Heart disease and stroke statistics-−2018 update: a report from the American Heart Association. Circulation. (2018) 137:e67–492. 10.1161/CIR.0000000000000558 29386200

[9] Zhang Q Zhang L Yang X Wan Y Jia J. The effects of exercise preconditioning on cerebral blood flow change and endothelin-1 expression after cerebral ischemia in rats. J Stroke Cerebrovasc Dis. (2014) 23:1696–702. 10.1016/j.jstrokecerebrovasdis.2014.01.016 24774439

[10] Tang F Barbacioru C Wang Y Nordman E Lee C Xu N. mRNA-Seq whole-transcriptome analysis of a single cell. Nat Methods. (2009) 6:377–82. 10.1038/nmeth.1315 19349980

[11] Alberter B Klein CA Polzer B. Single-cell analysis of CTCs with diagnostic precision: opportunities and challenges for personalized medicine. Expert Rev Mol Diagn. (2016) 16:25–38. 10.1586/14737159.2016.1121099 26567956

[12] Martijn J Schulz F Zaremba-Niedzwiedzka K Viklund J Stepanauskas R Andersson SGE. Single-cell genomics of a rare environmental alphaproteobacterium provides unique insights into Rickettsiaceae evolution. ISME J. (2015) 9:2373–85. 10.1038/ismej.2015.46 25848874 PMC4497978

[13] Lake BB Chen S Sos BC Fan J Kaeser GE Yung YC. Integrative single-cell analysis of transcriptional and epigenetic states in the human adult brain. Nat Biotechnol. (2018) 36:70–80. 10.1038/nbt.4038 29227469 PMC5951394

[14] Fuzik J Zeisel A Máté Z Calvigioni D Yanagawa Y Szabó G. Integration of electrophysiological recordings with single-cell RNA-seq data identifies neuronal subtypes. Nat Biotechnol. (2016) 34:175–83. 10.1038/nbt.3443 26689544 PMC4745137

[15] Johnson MB Walsh CA. Cerebral cortical neuron diversity and development at single-cell resolution. Curr Opin Neurobiol. (2017) 42:9–16. 10.1016/j.conb.2016.11.001 27888678 PMC5316371

[16] Gate D Saligrama N Leventhal O Yang AC Unger MS Middeldorp J. Clonally expanded CD8 T cells patrol the cerebrospinal fluid in Alzheimer's disease. Nature. (2020) 577:399–404. 10.1038/s41586-019-1895-7 31915375 PMC7445078

[17] Vanlandewijck M He L Mäe MA Andrae J Ando K Del Gaudio F. A molecular atlas of cell types and zonation in the brain vasculature. Nature. (2018) 554:475–80. 10.1038/nature25739 29443965

[18] Aibar S González-Blas CB Moerman T Huynh-Thu VA Imrichova H Hulselmans G. SCENIC: single-cell regulatory network inference and clustering. Nat Methods. (2017) 14:1083–6. 10.1038/nmeth.4463 28991892 PMC5937676

[19] Reddy PH Tonk S Kumar S Vijayan M Kandimalla R Kuruva CS. A critical evaluation of neuroprotective and neurodegenerative MicroRNAs in Alzheimer's disease. Biochem Biophys Res Communic. (2017) 483:1156–65. 10.1016/j.bbrc.2016.08.067 27524239 PMC5343756

[20] Vijayan M Reddy PH. Peripheral biomarkers of stroke: focus on circulatory microRNAs. Biochim Biophys Acta. (2016) 1862:1984–93. 10.1016/j.bbadis.2016.08.003 27503360 PMC5343760

[21] Selvamani A Williams MH Miranda RC Sohrabji F. Circulating miRNA profiles provide a biomarker for severity of stroke outcomes associated with age and sex in a rat model. Clin Sci. (2014) 127:77–89. 10.1042/CS20130565 24428837 PMC4386587

[22] Ryu CS Oh SH Lee KO Park HS An HJ Lee JY. MiR-10a, 27a, 34b/c, and 300 polymorphisms are associated with ischemic stroke susceptibility and post-stroke mortality. Life. (2020) 10:309. 10.3390/life10120309 33255549 PMC7760023

[23] Lidong D Zhanghong X Huawu M Xiaofang H Junhua G Kaifu K. Ischemia modified albumin and miR-126 play important role in diagnosis of posterior circulation transient ischemic attack and prediction of secondary cerebral infarction. Neurol India. (2021) 69:75–80. 10.4103/0028-3886.310100 33642274

[24] Vijayan M Kumar S Yin X Zafer D Chanana V Cengiz P. Identification of novel circulatory microRNA signatures linked to patients with ischemic stroke. Hum Mol Genet. (2018) 27:2318–29. 10.1093/hmg/ddy136 29701837 PMC6005038

[25] Liu Y Zhang J Han R Liu H Sun D Liu X. Downregulation of serum brain specific microRNA is associated with inflammation and infarct volume in acute ischemic stroke. J Clin Neurosci. (2015) 22:291–5. 10.1016/j.jocn.2014.05.042 25257664

[26] Mujaj B Bos D Muka T van der Lugt A Ikram MA Vernooij MW. Antithrombotic treatment is associated with intraplaque haemorrhage in the atherosclerotic carotid artery: a cross-sectional analysis of The Rotterdam Study. Eur Heart J. (2018) 39:3369–76. 10.1093/eurheartj/ehy433 30060115 PMC6148524

[27] Li P Zhu N Yi B Wang N Chen M You X. MicroRNA-663 regulates human vascular smooth muscle cell phenotypic switch and vascular neointimal formation. Circ Res. (2013) 113:1117–27. 10.1161/CIRCRESAHA.113.301306 24014830 PMC4537615

[28] Iaconetti C De Rosa S Polimeni A Sorrentino S Gareri C Carino A. Down-regulation of miR-23b induces phenotypic switching of vascular smooth muscle cells *in vitro* and *in vivo*. Cardiovasc Res. (2015) 107:522–33. 10.1093/cvr/cvv141 25994172

[29] Barr TL Conley Y Ding J Dillman A Warach S Singleton A. Genomic biomarkers and cellular pathways of ischemic stroke by RNA gene expression profiling. Neurology. (2010) 75:1009–14. 10.1212/WNL.0b013e3181f2b37f 20837969 PMC2942033

[30] Jickling GC Ander BP Zhan X Noblett D Stamova B Liu D. microRNA expression in peripheral blood cells following acute ischemic stroke and their predicted gene targets. PLoS ONE. (2014) 9:e99283. 10.1371/journal.pone.0099283 24911610 PMC4050059

[31] Stuart T Butler A Hoffman P Hafemeister C Papalexi E Mauck WM. comprehensive integration of single-cell data. Cell. (2019) 177:1888–902.e21. 10.1016/j.cell.2019.05.031 31178118 PMC6687398

[32] Lin W Wang Y Chen Y Wang Q Gu Z Zhu Y. Role of calcium signaling pathway-related gene regulatory networks in ischemic stroke based on multiple WGCNA and single-cell analysis. Oxid Med Cell Longev. (2021) 2021:1–35. 10.1155/2021/8060477 34987704 PMC8720592

[33] Chen Y Sun Y Xu Y Lin W-W Luo Z Han Z. Single-cell integration analysis of heterotopic ossification and fibrocartilage developmental lineage: endoplasmic reticulum stress effector Xbp1 transcriptionally regulates the notch signaling pathway to mediate fibrocartilage differentiation. Oxid Med Cell Longev. (2021) 2021:1–29. 10.1155/2021/7663366 34737845 PMC8563124

[34] Lin W-W Xu L-T Chen Y-S Go K Sun C Zhu Y-J. Single-cell transcriptomics-based study of transcriptional regulatory features in the mouse brain vasculature. Biomed Res Int. (2021) 2021:1–15. 10.1155/2021/7643209 34337051 PMC8324343

[35] Wu J Qin J Li L Zhang K Chen Y Li Y. Roles of the immune/methylation/autophagy landscape on single-cell genotypes and stroke risk in breast cancer microenvironment. Oxid Med Cell Longev. (2021) 2021:1–32. 10.1155/2021/5633514 34457116 PMC8397558

[36] Ritchie ME Phipson B Wu D Hu Y Law CW Shi W. Limma powers differential expression analyses for RNA-sequencing and microarray studies. Nucleic Acids Res. (2015) 43:e47. 10.1093/nar/gkv007 25605792 PMC4402510

[37] Feng W Yang J Song W Xue Y. Crosstalk between Heart Failure and Cognitive Impairment via hsa-miR-933/RELB/CCL21 Pathway. Biomed Res Int. (2021) 2021:1–16. 10.1155/2021/2291899 34595235 PMC8478533

[38] Han H Cho J-W Lee S Yun A Kim H Bae D. TRRUST v2: an expanded reference database of human and mouse transcriptional regulatory interactions. Nucleic Acids Res. (2018) 46:D380–6. 10.1093/nar/gkx1013 29087512 PMC5753191

[39] Yu G Wang L-G Han Y He Q-Y. clusterProfiler: an R package for comparing biological themes among gene clusters. OMICS J Integrat Biol. (2012) 16:284–7. 10.1089/omi.2011.0118 22455463 PMC3339379

[40] Subramanian A Tamayo P Mootha VK Mukherjee S Ebert BL Gillette MA. Gene set enrichment analysis: a knowledge-based approach for interpreting genome-wide expression profiles. Proc Natl Acad Sci. (2005) 102:15545–50. 10.1073/pnas.0506580102 16199517 PMC1239896

[41] Williamson-Reisdorph CM Quindry TS Tiemessen KG Cuddy J Hailes W Slivka D. Blood oxidative stress and post-exercise recovery are unaffected byhypobaric and hypoxic environments. J Sports Sci. (2021) 39:1356–65. 10.1080/02640414.2021.1872960 33423613

[42] Mohammadi SM Mohammadnejad D Hosseinpour Feizi AA Movassaghpour AA Montazersaheb S Nozad Charoudeh H. Inhibition of c-REL using siRNA increased apoptosis and decreased proliferation in pre-B ALL blasts: therapeutic implications. Leuk Res. (2017) 61:53–61. 10.1016/j.leukres.2017.08.012 28892661

[43] Yang W Lu Z Zhi Z Liu L Deng L Jiang X. High-throughput transcriptome-Seq and small RNA-Seq reveal novel functional genes and microRNAs for early embryonic arrest in humans. Gene. (2019) 697:19–25. 10.1016/j.gene.2018.12.084 30776465

[44] Li Y-Y Guo J-H Liu Y-Q Dong J-H Zhu C-H. PPARγ activation-mediated Egr-1 inhibition benefits against brain injury in an experimental ischaemic stroke model. J Stroke Cerebrovasc Dis. (2020) 29:105255. 10.1016/j.jstrokecerebrovasdis.2020.105255 32992165

[45] Yan S-F Fujita T Lu J Okada K Shan Zou Y Mackman N. Egr-1, a master switch coordinating upregulation of divergent gene families underlying ischemic stress. Nat Med. (2000) 6:1355–61. 10.1038/82168 11100120

[46] Okada M Fang Yan S Pinsky DJ. Peroxisome proliferator-activated receptor-γ (PPAR-γ) activation suppresses ischemic induction of Egr-1 and its inflammatory gene targets. FASEB J. (2002) 16:1861–8. 10.1096/fj.02-0503com 12468449

[47] Tureyen K Brooks N Bowen K Svaren J Vemuganti R. Transcription factor early growth response-1 induction mediates inflammatory gene expression and brain damage following transient focal ischemia. J Neurochem. (2008) 105:1313–24. 10.1111/j.1471-4159.2008.05233.x 18208539 PMC2603292

[48] Peyrou M Ramadori P Bourgoin L Foti M. PPARs in liver diseases and cancer: epigenetic regulation by microRNAs. PPAR Res. (2012) 2012:1–16. 10.1155/2012/757803 23024649 PMC3449131

[49] Zhao C Zhang T Shi Z Ding H Ling X. MicroRNA-518d regulates PPARα protein expression in the placentas of females with gestational diabetes mellitus. Mol Med Rep. (2014) 9:2085–90. 10.3892/mmr.2014.2058 24639097

[50] Lee SE Yang H Son GW Park HR Cho J-J Ahn H-J. Identification and characterization of MicroRNAs in acrolein-stimulated endothelial cells: implications for vascular disease. Biochip J. (2015) 9:144–55. 10.1007/s13206-015-9303-3

[51] Shi J Ren Y Liu Y Cheng Y Liu Y. Circulating miR-3135b and miR-107 are potential biomarkers for severe hypertension. J Hum Hypertens. (2021) 35:343–50. 10.1038/s41371-020-0338-0 32327699

[52] Wang F Li Z Zhao M Ye W Wu H Liao Q. Circulating miRNAs miR-574-5p and miR-3135b are potential metabolic regulators for serum lipids and blood glucose in gestational diabetes mellitus. Gynecol Endocrinol. (2021) 37:665–71. 10.1080/09513590.2021.1908990 34126831

[53] Liu W Ling S Sun W Liu T Li Y Zhong G. Circulating microRNAs correlated with the level of coronary artery calcification in symptomatic patients. Sci Rep. (2015) 5:16099. 10.1038/srep16099 26537670 PMC4633594

[54] Naito H Hosomi N Kuzume D Nezu T Aoki S Morimoto Y. Increased blood pressure variability during the subacute phase of ischemic stroke is associated with poor functional outcomes at 3 months. Sci Rep. (2020) 10:811. 10.1038/s41598-020-57661-z 31964961 PMC6972830

[55] Kissela BM Khoury J Kleindorfer D Woo D Schneider A Alwell K. Epidemiology of ischemic stroke in patients with diabetes. Diabetes Care. (2005) 28:355–9. 10.2337/diacare.28.2.355 15677792

[56] Boden-Albala B Sacco RL Lee H-S Grahame-Clarke C Rundek T Elkind MV. Metabolic syndrome and ischemic stroke risk: northern Manhattan study. Stroke. (2008) 39:30–5. 10.1161/STROKEAHA.107.496588 18063821 PMC2677015

[57] Alves-Junior L Niemeier S Hauenschild A Rehmsmeier M Merkle T. Comprehensive prediction of novel microRNA targets in *Arabidopsis thaliana*. Nucleic Acids Res. (2009) 37:4010–21. 10.1093/nar/gkp272 19417064 PMC2709567

[58] Zhang Z-L Song Y Li F Huang Q-B. Bimodal distribution of nuclear factor-κB activation and expression of subunits in experimental models of intracerebral hemorrhage *in vivo*. J Stroke Cerebrovasc Dis. (2019) 28:821–9. 10.1016/j.jstrokecerebrovasdis.2018.11.028 30558860

[59] Sun J Huang P Liang J Li J Shen M She X. Cooperation of Rel family members in regulating Aβ1-40-mediated pro-inflammatory cytokine secretion by retinal pigment epithelial cells. Cell Death Dis. (2017) 8:e3115. 10.1038/cddis.2017.502 29022897 PMC5682668

[60] Deng Y Xiong D Yin C Liu B Shi J Gong Q. Icariside II protects against cerebral ischemia–reperfusion injury in rats via nuclear factor-κB inhibition and peroxisome proliferator-activated receptor up-regulation. Neurochem Int. (2016) 96:56–61. 10.1016/j.neuint.2016.02.015 26939761

[61] Mo Y Sun Y-Y Liu K-Y. Autophagy and inflammation in ischemic stroke. Neural Regen Res. (2020) 15:1388. 10.4103/1673-5374.274331 31997797 PMC7059569

[62] Wu KK Kuo C-C Yet S-F Lee C-M Liou J-Y. 5-methoxytryptophan: an arsenal against vascular injury and inflammation. J Biomed Sci. (2020) 27:79. 10.1186/s12929-020-00671-w 32635910 PMC7341587

[63] Santiago JA Scherzer CR Potashkin JA. Network analysis identifies SOD2 mRNA as a potential biomarker for Parkinson's Disease. PLoS ONE. (2014) 9:e109042. 10.1371/journal.pone.0109042 25279756 PMC4184821

[64] Schramm F Kern A Barthel C Nadaud S Meyer N Jaulhac B. Microarray analyses of inflammation response of human dermal fibroblasts to different strains of Borrelia burgdorferi Sensu Stricto. PLoS ONE. (2012) 7:e40046. 10.1371/journal.pone.0040046 22768217 PMC3386942

[65] Kang L Dai C Lustig ME Bonner JS Mayes WH Mokshagundam S. Heterozygous SOD2 deletion impairs glucose-stimulated insulin secretion, but not insulin action, in high-fat–fed mice. Diabetes. (2014) 63:3699–710. 10.2337/db13-1845 24947366 PMC4207395

[66] Singh BK Kumar A Ahmad I Kumar V Patel DK Jain SK. Oxidative stress in zinc-induced dopaminergic neurodegeneration: implications of superoxide dismutase and heme oxygenase-1. Free Rad Res. (2011) 45:1207–22. 10.3109/10715762.2011.607164 21777051

[67] Tanaka K Nagata E Suzuki S Dembo T Nogawa S Fukuuchi Y. Immunohistochemical analysis of cyclic AMP response element binding protein phosphorylation in focal cerebral ischemia in rats. Brain Res. (1999) 818:520–6. 10.1016/s0006-8993(98)01263-3 10082840

[68] Lake RJ Tsai P-F Choi I Won K-J Fan H-Y. RBPJ, the major transcriptional effector of notch signaling, remains associated with chromatin throughout mitosis, suggesting a role in mitotic bookmarking. PLoS Genet. (2014) 10:e1004204. 10.1371/journal.pgen.1004204 24603501 PMC3945225

[69] Liu Z-J Tan Y Beecham GW Seo DM Tian R Li Y. Notch activation induces endothelial cell senescence and pro-inflammatory response: implication of Notch signaling in atherosclerosis. Atherosclerosis. (2012) 225:296–303. 10.1016/j.atherosclerosis.2012.04.010 23078884 PMC3502717

[70] Meng L Bai Z He S Mochizuki K Liu Y Purushe J. The notch ligand DLL4 defines a capability of human dendritic cells in regulating Th1 and Th17 differentiation. JI. (2016) 196:1070–80. 10.4049/jimmunol.1501310 26712946 PMC4930627

[71] Harrison DA. The JAK/STAT pathway. Cold Spring Harbor Perspect Biol. (2012) 4:a011205. 10.1101/cshperspect.a011205 22383755 PMC3282412

